# Malignant primary pericardial mesothelioma presenting as effusive constrictive pericarditis: a case report study

**DOI:** 10.1186/s13019-021-01684-8

**Published:** 2021-10-13

**Authors:** Amir Savarrakhsh, Azin Vakilpour, Sam Zeraatian-Nejad Davani, Mahyar Daskareh, Mahdieh Morsaghian, Arsalan Salari, Seyedeh Fatemeh Mirrazeghi

**Affiliations:** 1 Cardiovascular Diseases Research Center, Department of Cardiology, Heshmat Hospital School of Medicine, Guilan University of Medical Sciences, 15 Khordad Street, District 2, Rasht, Guilan Province Iran; 2 Department of Cardiology, Rasool Akram General Hospital, Iran University of Medical Sciences, Tehran, Iran; 3 Department of Radiology, Ziaeian Hospital, Tehran University of Medical Sciences, Tehran, Iran

**Keywords:** Case report, Cardiac tumors, Constrictive pericarditis, Primary pericardial mesothelioma

## Abstract

**Background:**

Primary pericardial mesothelioma (PPM) is a rare malignancy with a high prevalence of mortality. The diagnosis is usually challenging using a variety of imaging modalities and invasive procedures and is generally performed at the later stages of the disease or in autopsy. This case study points to an unconventional presentation of PPM and the challenges in diagnosing this rare mortal malignancy.

**Case presentation:**

This study presents a 44-year-old woman with no remarkable medical history with an initial diagnosis of effusive constrictive pericarditis at first hospitalization. Imaging evaluations, including transthoracic echocardiography and chest computed tomography scan, demonstrated visible thickened pericardium, pericardial effusion, and mass-like lesions in pericardium and mediastinum. The definite diagnosis of primary pericardial mesothelioma was established after pericardiectomy and histopathology examinations. Chemotherapy with pemetrexed and carboplatin was administrated to the patient, and she has been through four cycles of chemotherapy with no complications to date.

**Conclusion:**

Constrictive pericarditis is an uncommon presentation of PPM. Due to the high mortality rate and late presentation, difficulties and uncertainties in diagnosis, being aware of this rare malignant entity in different cardiac manifestations, particularly when there is no clear explanation or response to treatment in such conditions, is highly important.

**Supplementary Information:**

The online version contains supplementary material available at 10.1186/s13019-021-01684-8.

## Background

Primary cardiac neoplasms are oncology rarities with the prevalence of 0.001–0.056% and account for 0.3 to 0.7% of all cardiac cancers. Only 25% of primary cardiac neoplasms are malignant [1]. Among cardiac tumors, primary neoplasms arising from pericardium with the estimated prevalence of 0.001–0.007% are exceptionally infrequent [2]. Meanwhile, Primary pericardial mesothelioma (PPM) with a prevalence rate of less than 0.002% is known as a rare malignancy [3]. Malignant mesothelioma is a neoplasm arising from the mesothelial surface within cavities such as plural cavity, peritoneum, and rarely pericardium and tunica vaginalis testis [4]. PPM is a highly invasive tumor and at the time of diagnosis is usually at a late stage. The survival rate is low, and the average life expectancy after diagnosis is reported between 3 to 10 months [5].

## Case presentation

A 44-year-old woman introduced herself to the emergency department in November 2020, complaining of epigastric pain, progressive dyspnea, nausea, premature satiety, and anorexia. She also stated that she had been suffering from deterioration of her general condition accompanied by weakness, fatigue, lethargy, dyspnea (NYHA class ΙΙ), and a 12 kg unwanted weight loss within 2 years. No cough, sputum, orthopnea, and prior cardiac diseases were claimed. Previous medical history included multiple muscle problems and neck pain which had been treated with a diagnosis of Fibromyalgia. No radiation, chest surgery, or trauma history and no history of diagnosed cancer in her family were mentioned. The patient was a non-smoker and had no history of asbestos exposure. Medication history included Gabapentin 100 mg twice daily and oral contraceptive pills.

Physical examination revealed a blood pressure of 112/80, heart rate of 100 beats/min, respiratory rate of 16 breaths/min, and a temperature of 36.5 °C.

O2 saturation was 94%. The heart sounds were normal with a regular rhythm, and no murmur was found. In lung auscultation, no remarkable finding was detected. The patient did not have elevated JVP as well as abdominal ascites and edema in the lower extremities. In laboratory findings, the CBC revealed anemia (Hb: 8.0 g/dl) and thrombocytosis (PLT: 609 000 U/L). The patient's blood chemistry showed elevated inflammatory markers level. (ESR: 101 mg/dl, CRP: 24 mg/dl, WBC: 17.2 billion/l) along with an increased lactate dehydrogenase level (LDH: 1757 U/L).

**Fig. 1:**
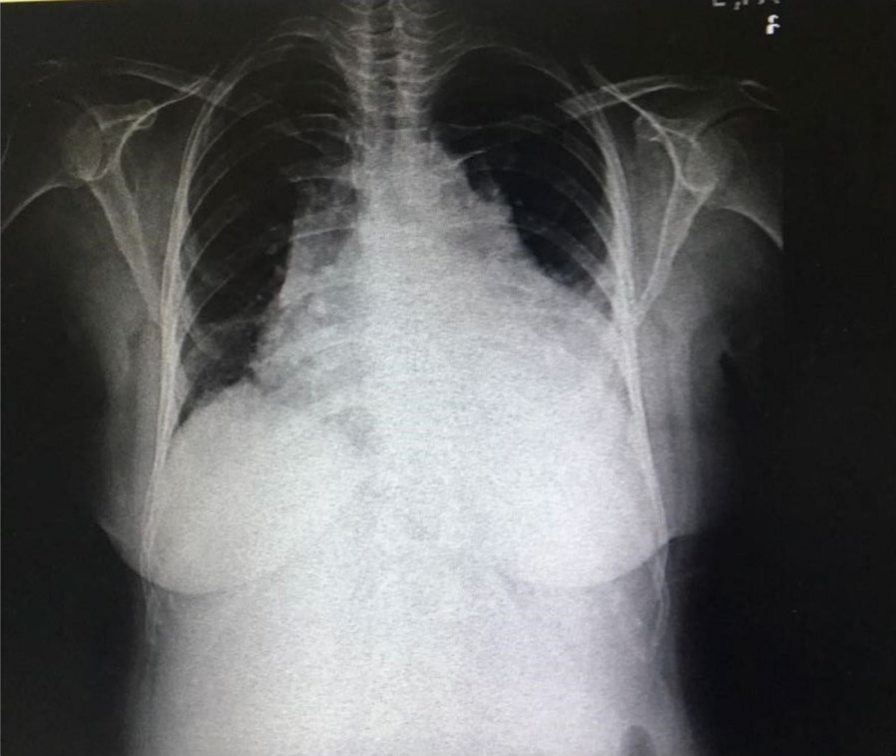
Initial chest radiography of the patient. Chest radiography showing widened mediastinum, cardiomegaly, and left pleural effusion

Chest X-ray on admission revealed a cardiothoracic index of > 50%, widened mediastinum, and pleural effusion in the left hemithorax (Fig. 1). First Electrocardiography showed T inversion in leads Ι and avL, but the troponin level was normal. Thereafter, the patient was referred to our hospital, which is the only referral center for cardiovascular diseases.

**Fig. 2:**
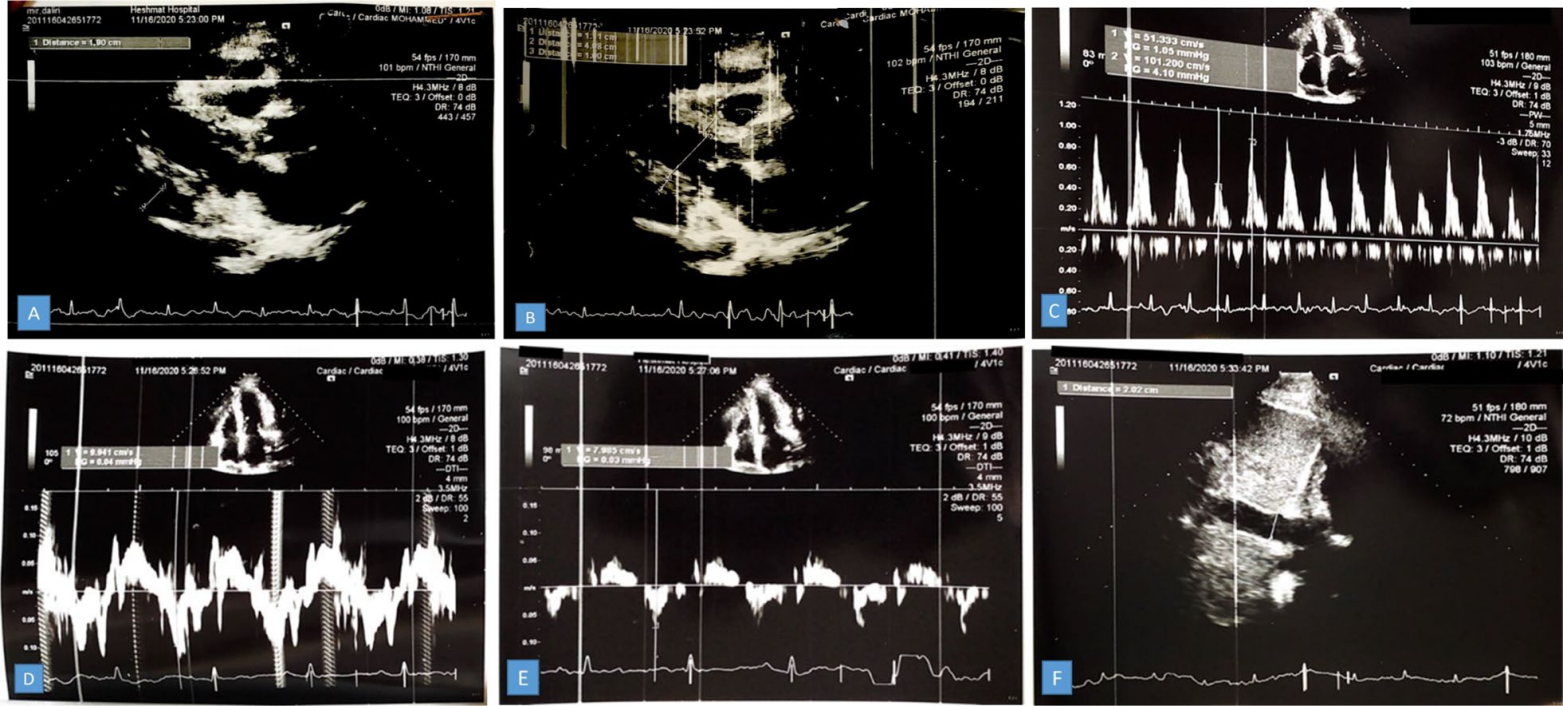
Echocardiography demonstrating Thickened pericardium and effusion in parasternal long-axis view (**A**, **B**), Exaggerated respiratory variation at the level of Mitral Valve **(C)**, Annulus Reversus **(D**, **E)**, normal IVC (20 mm) with reduced respiratory variation **(F)**

A transthoracic echocardiogram was performed and demonstrated normal left ventricular (LV) size with mild systolic dysfunction (LVEF: 45–50%), moderate LV diastolic dysfunction with annulus reversus (E’septal > E’lat), normal inferior vena cava (IVC) with reduced respiratory collapse, normal right ventricle (RV) size with moderate systolic dysfunction (RVSM 8 cm/s) and left atrium enlargement. Moderate mitral regurgitation, no mitral stenosis with significant MIF respiratory change, and at least moderate tricuspid regurgitation (TRG: 36 mmHg) were also reported. Additionally, Visible thickened pericardium with an obvious thickness (in size 24 mm in anterior RVOT) and mixed pericardial effusion (size 19 mm in posterior LV) and visible mass-like lesion in size approximately 42 mm in inferolateral RV were declared in the echocardiogram report (Fig. 2).

All of the above findings were consistent with constrictive pericarditis. Thereafter, the Patient was referred to a tertiary hospital in order to further investigations. Computed tomography was performed and demonstrated cardiomegaly, mild pleural effusion on the left side, and a nodular infiltrative mass in anterior and middle mediastinum accompanied by pulmonary vessels enhancements and pericardial involvement. Multiple lymph nodes in mediastinum and multiple hypoechoic lesions in the liver were also reported. In addition, several multi-focal and multi-lobar ground-glass opacities with peripheral dominancy in both lungs were seen, which considering the Covid-19 pandemic, was suggestive of Covid-19 viral pneumonia. Pleural fluid cytology evaluation was negatively malignant. The patient underwent the Covid-19 RT-PCR test via oropharyngeal and nasopharyngeal sampling, and the result was positive. Then, according to the consultant infectious disease specialist, based on her clinical symptoms and examinations (no fever, no cough, no sputum, no myalgia, or severe weakness, normal respiratory rate and normal O2 saturation ≥ 94%), patient was isolated and underwent conservative therapy and supportive care for the COVID-19 infection during hospitalization. The patient successfully recovered from COVID-19 infection.

Radiological consultation for biopsy of the lesions (mediastinal mass or liver mass) was requested, and due to the thickness of the pericardium, pericardial biopsy was recommended.

The patient was decided to undergo pericardiectomy surgery. Patient with severe adhesive pericarditis and multiple effusive pericardial effusion underwent midline sternotomy with oscillator saw, and all adhesions released from heart epicardium in a delicate manner from inferior side RV up to ascending aorta as well as pulmonary trunk and was sent for pathology examination due to its very bizarre appearance and consistency. According to the surgery report, during the procedure, abnormal appearance of the pericardium and macroscopic infiltrations to heart muscle were visible. Left ventricular mass resection and thymus gland biopsy were also performed. Additional files 1–9 show the surgery procedure, releasing adhesions from epicardium, and mass resection.

### Histopathological examination

Specimens were sent to histopathology evaluation in 2 containers labeled as thymus gland and pericardial mass. Thymus gland specimen in macroscopic examination consisted of multiple fragments of fibro-fatty tissue, TM: 5 × 5 × 4 cm with foci of firm consistency. On sections, foci of firm gray, creamy areas were seen on the surface; the largest measure was 1.5 cm in greatest diameter. No mass was identified grossly.

**Fig. 3:**
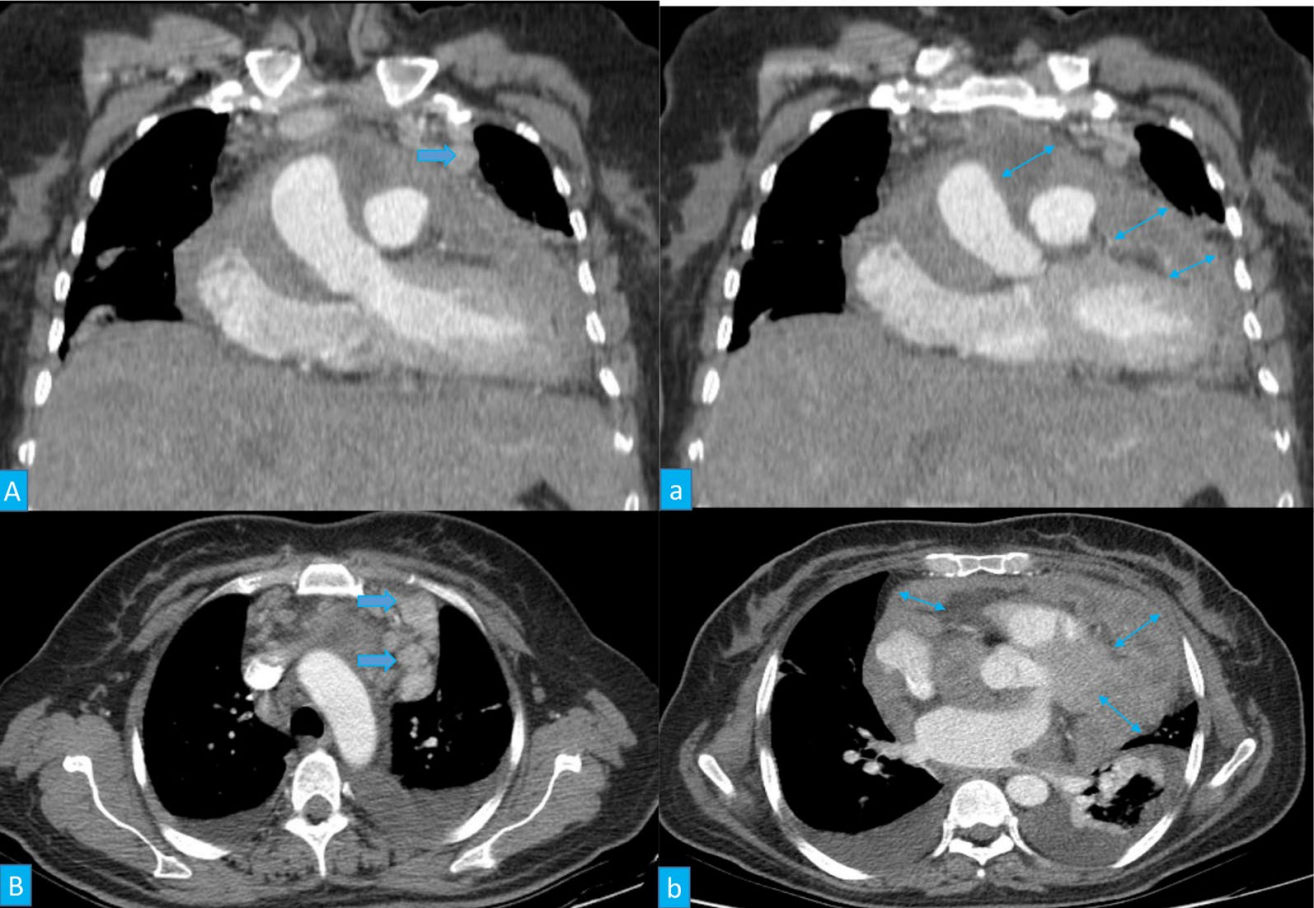
Two reconstructed coronal computed tomography images (**A**, a) and thin section axial computed tomography images (**B**, b) revealed some large enhancing nodes in the left anterior mediastinum (arrows), and nodular thickening of the pericardium (double arrows) with irregularity and enhancement associated with effusion compressing heart chambers

The pericardial mass consisted of multiple fragments of fibro-fatty tissue with foci of firm consistency TM: 7 × 7 × 3 cm. On sections, foci of creamy rubbery to firm area were seen measuring about 4 × 4 × 2 cm. In the microscopic examination, the section from pericardial lesion showed proliferation of mesothelial cells with a tubulopapillary architecture and few solid areas infiltrated between muscle handles and was in intimate contact with adipocyte. The tumor cells were characterized by enlarged vesicular to hyperchromatic nuclei, occasional prominent nucleoli, and moderate amount of amphophilic cytoplasm. No necrosis was identified (Fig. 3).

**Fig. 4:**
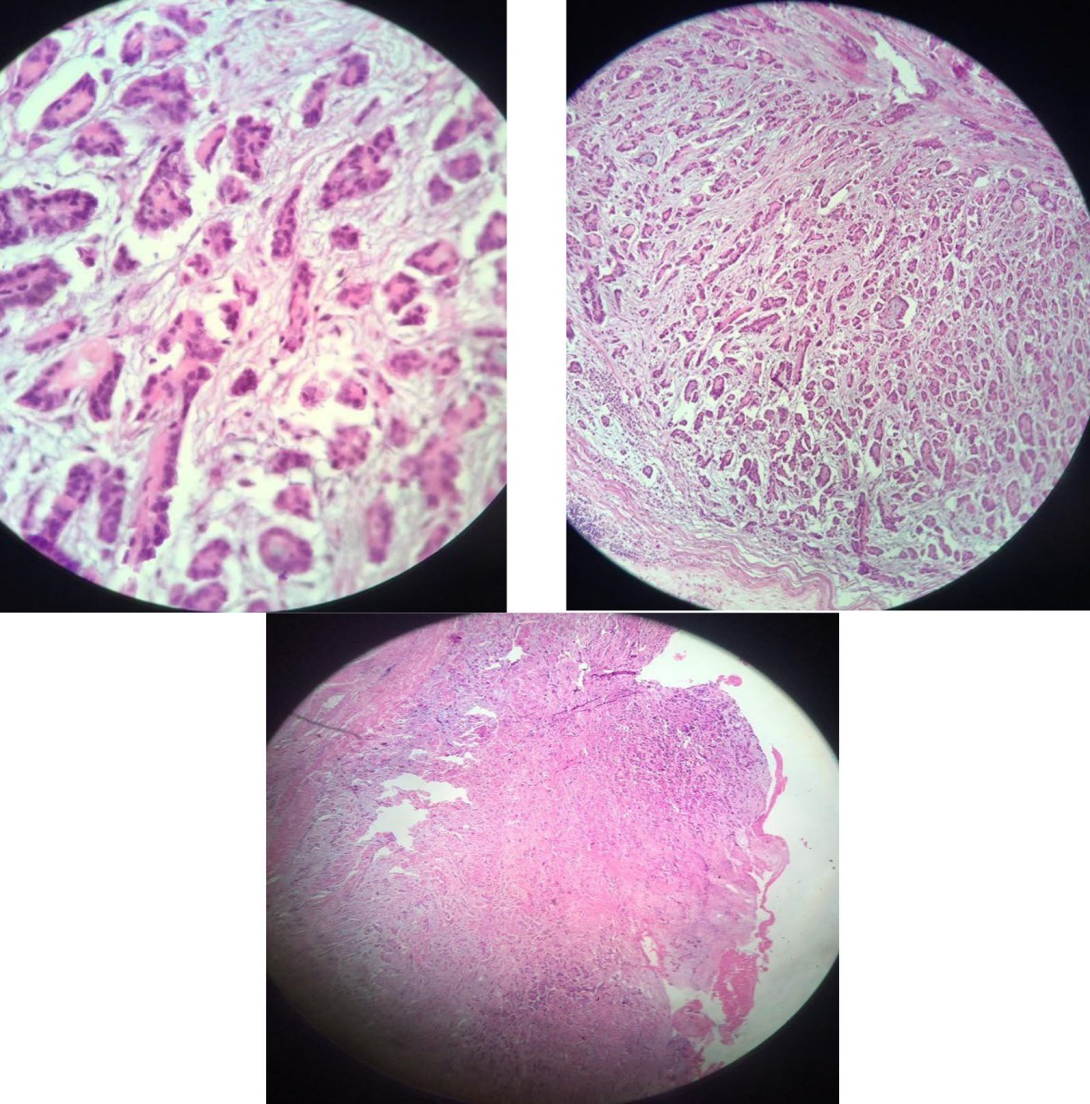
Histopathology examination of post-pericardiectomy specimen revealing proliferation of mesothelial cells with infiltrative and tubule-papillary architecture

Overall, histopathological examination of the pericardial lesion demonstrated malignant epithelioid mesothelioma, and the tumor size was reported 4 cm in greatest diameter. No vascular invasion was noted. Also, the thymus gland showed malignant mesothelioma on the surface, and no clear-cut evidence of invasion to the thymus gland by tumor was detected (Fig. 4).

### Treatment

Thereafter, due to the massive extension of the tumor to the mediastinum and heart as well as the detection of hypoechoic lesions in the liver in CT-scan suggesting distant metastasis, chemotherapy with pemetrexed 500 mg/m^2^ in combination with carboplatin were administrated to the patient. To date, she has been through four cycles of chemotherapy with no complications, and she has been asymptomatic and in remission so far.

## Discussion

Our case points to an unconventional presentation of PPM as well as the challenges in diagnosing this rare mortal malignancy. The patient was presented with unspecific manifestations, and although it took about 2 years from the onset of symptom to PPM diagnosis, constrictive pericarditis, which is considered a less common presentation of PPM, was diagnosed at first hospitalization. Following several diagnostic approaches such as transthoracic echocardiography and CT scan evidence suggesting a malignant entity, she was quickly referred to a facilitated tertiary hospital for surgery, histopathology examinations, and further investigation. The majority of cardiac malignancies are secondary due to metastasis from other organs’ neoplasms, primarily lung, breast, melanomas, and hematologic cancers [6, 7]. It has been previously reported that pericardial mesothelioma represents for 0.7% of all malignant mesotheliomas [8]. The causes and risk factors are still unknown [4, 9]. Some studies have suggested a possible relationship between exposure to asbestos and primary pericardial malignancy, but no definite correlation has been established yet [7].

PPM has been reported to be more prevalent in men and can occur at any age, but mostly is diagnosed between the ages of 50 to 70 with a median age of 46 years [5]. It can show several manifestations, among which non-specific presentations, including constitutional symptoms, are frequent. Other manifestations such as acute pericarditis, cardiac tamponade, constrictive pericarditis, and heart failure presentations may also be present [4, 5, 7]. Malignant pericardial mesothelioma can be misdiagnosed with cardiovascular diseases such as coronary heart disease, cardiomyopathy, tuberculosis pericarditis, and pericardial metastatic tumors due to non-specific manifestations as well as rarity and uncertainties in diagnosis. Furthermore, signs and symptoms can be easily masked using various cardiac therapies [9].

PPM is known to be a highly lethal and metastatic disease owing to late presentation and poor response to treatment. The diagnosis of pericardial mesothelioma is usually late and at the end stages of the disease via surgery or autopsy [3, 4, 9]. Laboratory information is an integral part of the diagnosis, prognosis, and treatment of the disease. Notable findings include increased inflammatory markers level as well as anemia and low serum albumin level [9]. Of note, a vast body of literature has evaluated the role of pretreatment LDH level as a convenient prognostic factor in patients with malignant mesothelioma [10–13]. A meta-analysis conducted by Yi et al. demonstrated a negative relationship between LDH level before initiating treatment and poor survival. They suggested that pretreatment LDH level can be valuable as a predictor of prognosis in such patients [14].

Multiple diagnostic imaging measures are usually required to diagnose PPM [6, 7]. Echocardiography is commonly used, but its sensitivity has been reported to be low [5]. Common echocardiographic findings in pericardial malignancies are pericardial effusion, cardiac tamponade, pericardial thickening, and mass lesions [9].

Other diagnostic tools include computed tomography (CT), magnetic resonance imaging (MRI), and FDG-PET scan, which are very valuable for evaluating the tumor size, tumor location, extension to vicinal structures, and also staging of cancer [2, 7]. However, the definite diagnosis is based on histopathology, and pericardial biopsy is mostly necessary due to the limited roles of cytology and difficulties in pericardial effusion aspiration [9]. Pericardial mesothelioma usually shows a poor response to treatment. Surgery has remained the main treatment option so far; however, it is not performable in all patients. In some cases, including small size tumors and in the absence of a diffuse extension, surgical interventions and resection may be curative and also helpful in reducing effusion and constriction [2, 7]. A palliative strategy based on radiotherapy, chemotherapy, and surgery is widely used [4, 6, 15]. Recently, a combination chemotherapy regimen using different agents has been told to be possibly effective [9], for instance; according to some previous studies, chemotherapy using pemetrexed (a synthetic pyrimidine-based antifolate) with platin compounds such as cisplatin or carboplatin has shown to be effective in improving patient's survival time, but the results were not as impressive as in pleural mesothelioma [3, 9, 16].

## Conclusion

Our patient was presented with non-specific symptoms and electrocardiography changes accompanied by elevated LDH and inflammatory markers. After multimodality imaging, effusive constrictive pericarditis, which is considered an unconventional presentation of PPM, was diagnosed. Therefore, although PPM is known as a rare and highly lethal disease due to itlate presentation and uncertainties in diagnosis, it is of importance to be aware of this condition in various cardiac manifestations, including pericardial effusion, cardiac tamponade, patients presenting with clinical and imaging findings consistent with constrictive pericarditis, and even heart failure presentations, particularly when there is no clear explanation and response to treatment in such conditions.

## Supplementary Information

**Additional file 1.** Additional movie files show the surgery procedure, releasing adhesions from heart epicardium as well as mass resection.

**Additional file 2.** Showing the surgery procedure.

**Additional file 3.** Showing the surgery procedure.

**Additional file 4.** Showing the surgery procedure.

**Additional file 5.** Showing the surgery procedure.

**Additional file 6.** Showing the surgery procedure.

**Additional file 7.** Showing the surgery procedure.

**Additional file 8.** Showing the surgery procedure.

**Additional file 9.** Showing the surgery procedure.

## Data Availability

Not applicable.

## Abbreviations

PPM: Primary pericardial mesothelioma
CBC: Complete blood count
Hb: Hemoglobin
WBC: White blood cell
PLT: Platelets
LDH: Lactate dehydrogenase
LVEF: Left ventricular ejection fraction
RV: Right ventricle
IVC: Inferior vena cava
RVOT: Right ventricular outflow tract
TRG: Tricuspid regurgitation gradient
CT: Computed tomography
RT-PCR: Real-time reverse transcription-polymerase chain reaction
MRI: Magnetic resonance imaging
FDG-PET: Fluorodeoxyglucose-positron emission tomography

## References

[1] Leja MJ, Shah DJ, Reardon MJ (2011). Primary cardiac tumors. Tex Heart Inst J.

[2] Barroso AS, Leite S, Friões F, Vasconcelos M, Azevedo D, Baldaia H, et al. Pericardial mesothelioma presenting as a suspected ST-elevation myocardial infarction. Revista Portuguesa de Cardiologia (English Edition). 2017;36(4):307. e1–e5. 10.1016/j.repc.2016.03.014 28343785

[3] Kim JS, Lim SY, Hwang J, Kang EJ, Choi YJ (2017). A case report of primary pericardial malignant mesothelioma treated with Pemetrexed and Cisplatin. J Korean Med Sci.

[4] Godar M, Liu J, Zhang P, Xia Y, Yuan Q. Primary pericardial mesothelioma: a rare entity. Case reports in oncological medicine. 2013;2013, 1. 10.1155/2013/283601 PMC3697233 23840993

[5] Tajjiou M, Wild W, Sayed N, Flauaus A, Divo M, Schwarzbach M. Primary pericardial mesothelioma, which was veiled by a pleural empyema: a case report and review. Case Rep Surg. 2019. 10.1155/2019/2896810 PMC6755280 31612092

[6] Butz T, Faber L, Langer C, Körfer J, Lindner O, Tannapfel A (2009). Primary malignant pericardial mesothelioma-a rare cause of pericardial effusion and consecutive constrictive pericarditis: a case report. J Med Case Rep.

[7] Suman S, Schofield P, Large S. Primary pericardial mesothelioma presenting as pericardial constriction: a case report. Heart. 2004;90(1):e4-e. 10.1136/heart.90.1.e4 PMC1767997 14676267

[8] Mensi C, Romano A, Berti A, Dore R, Riboldi L (2017). A second case of pericardial mesothelioma mimicking systemic lupus erythematosus in the literature in over 30 years: a case report. J Med Case Rep.

[9] Cao S, Jin S, Cao J, Shen J, Zhang H, Meng Q (2018). Malignant pericardial mesothelioma. Herz.

[10] Suzuki H, Hirashima T, Kobayashi M, Okamoto N, Matsuura Y, Tamiya M (2012). Prognostic factors in malignant pleural mesothelioma: a retrospective study. Intern Med.

[11] Kataoka Y, Yamamoto Y, Otsuki T, Shinomiya M, Terada T, Fukuma S (2015). A new prognostic index for overall survival in malignant pleural mesothelioma: the rPHS (regimen, PS, histology or stage) index. Jpn J Clin Oncol.

[12] Abakay O, Tanrikulu AC, Palanci Y, Abakay A (2014). The value of inflammatory parameters in the prognosis of malignant mesothelioma. J Int Med Res.

[13] Suzuki H, Asami K, Hirashima T, Okamoto N, Yamadori T, Tamiya M (2014). Stratification of malignant pleural mesothelioma prognosis using recursive partitioning analysis. Lung.

[14] Zhuo Y, Lin L, Wei S, Zhang M (2016). Pretreatment elevated serum lactate dehydrogenase as a significant prognostic factor in malignant mesothelioma: a meta-analysis. Medicine.

[15] Fujita K, Hata M, Sezai A, Minami K (2014). Three-year survival after surgery for primary malignant pericardial mesothelioma: report of a case. Surg Today.

[16] Cao S, Jin S, Cao J, Shen J, Hu J, Che D (2015). Advances in malignant peritoneal mesothelioma. Int J Colorectal Dis.

